# Quaternary climate change and habitat preference shaped the genetic differentiation and phylogeography of *Rhodiola* sect. *Prainia* in the southern Qinghai–Tibetan Plateau

**DOI:** 10.1002/ece3.5406

**Published:** 2019-06-30

**Authors:** Zi‐Meng Wang, Shi‐Yong Meng, Guang‐Yuan Rao

**Affiliations:** ^1^ School of Life Sciences Peking University Beijing China

**Keywords:** habitat preference, in situ survival, *Rhodiola* sect. *Prainia*, *tabula rasa*, the Qinghai–Tibetan Plateau

## Abstract

There are two long‐standing biogeographic hypotheses regarding the glacial survival of plant species in the Qinghai–Tibetan Plateau (QTP): the in situ survival hypothesis and the *tabula rasa* hypothesis. We tested these two hypotheses in a phylogeographic study of *Rhodiola* sect. *Prainia*, a monophyletic section with ecologically divergent lineages. Molecular data from the nuclear internal transcribed spacer, six plastid markers and 13 nuclear microsatellite loci were analyzed for 240 individuals from 19 populations of this section. Environmental data were used to analyze the niches of major phylogenetic lineages within this section and to model changes in their distributions since the Last Glacial Maximum (LGM). We found that *Rhodiola* sect. *Prainia* consists of three evolutionary lineages: all populations of *R. stapfii*, *R. prainii* populations at the southern edge of the QTP, and *R. prainii* populations in the interior part of the QTP. During the LGM, the survival of *R. prainii* in the interior part of the QTP corresponded with the in situ survival hypothesis, while *R. stapfii* most probably survived the LGM in a manner corresponding with the *tabula rasa* hypothesis. The evolutionary history of different lineages of this section was shaped by topography, climate change, and lineage‐specific habitat preferences.

## 1 INTRODUCTION

The current distribution of plant species has been shaped by Quaternary climate oscillations (Hewitt, 2000). Glacial survival and postglacial dynamics have been intensively studied worldwide, and the results generally fall into two alternative hypotheses: (a) in situ survival (the *nunatak* hypothesis) and (b) postglacial recolonization (the *tabula rasa* hypothesis; Stewart, Lister, Barnes, & Dalén, 2010; Qiu, Fu, & Comes, 2011). There is a long‐standing debate regarding how arctic–alpine species survived glaciations (Hewitt, 2004). The *tabula rasa* hypothesis is supported by the majority of molecular studies of arctic–alpine species and some evidence of fossil studies (reviewed by Brochmann, Gabrielsen, Nordal, Landvik, & Elven, 2003; Tzedakis, Emerson, & Hewitt, 2013), whereas the in situ survival hypothesis receives supports primarily from fossil data (reviewed by Willis & Andel, 2004) and recent molecular studies (Quinzin, Normand, Dellicour, Svenning, & Mardulyn, 2017; Westergaard et al., 2011). To better understand the causes of the current distribution patterns of alpine species, *that is*, the two abovementioned hypotheses, influence of topography and climate changes on the distribution of alpine species should be considered, with a particular focus on taxon‐specific characteristics (Papadopoulou & Knowles, 2016).

The Qinghai–Tibetan Plateau (QTP) consists of many mountains, flatlands, and valleys, and it provides a wide range of potential refugia and geographical barriers for testing hypotheses regarding glacial survival. The timing and extent of glaciations in the QTP during the Last Glacial Maximum (LGM) were still disputable, but it is generally agreed that there was no ice sheet covering the entire plateau and the valleys of the Yarlung Zangbo River were mostly unglaciated (Owen & Dortch, 2014). Fossil pollen records in the QTP reveal that vegetation zones have moved and expanded from the southeast to the northwest, from the edge of the plateau to the hinterland, and from low elevations to high elevations during the Holocene (Hou, Yang, Cao, Chongyi, & Wang, 2015). These changes in plant distribution allowed both postglacial colonization from peripheral areas and in situ survival via vertical movement along mountain slopes. Many alpine trees and shrubs occurring in the QTP, including *Potentilla fruticosa* (Li, Shimono, Shen, & Tang, 2010), *Potentilla glabra* (Wang, Ikeda, Liu, Wang & Liu 2009), the *Juniperus tibetica* complex (Opgenoorth et al., 2010), *Spiraea alpina* (Zhang et al., 2012), and a lineage of *Hippophae tibetana* (Wang et al., 2010), probably survived in situ during Quaternary glaciations. In contrast, some other alpine trees and shrubs, including *Juniperus przewalskii* (Li, Zhang, Liu, Källman, & Lascoux, 2011; Zhang, Chiang, George, Liu, & Abbott, 2005), *Picea crassifolia* (Meng et al., 2007), and *Tsuga dumosa* (Cun & Wang, 2010), migrated through these barriers and recolonized the QTP. The herb species of the QTP show similar differences in their demographic histories. For example, four subnival herbs survived locally in the southern QTP (Luo et al., 2016), while the alpine herbs *Metagentiana striata* (Chen et al., 2008) and *Pedicularis longiflora* (Yang, Li, Ding, & Wang, 2008) recolonized the QTP from its southeastern edge.

Previous studies of various taxa in the QTP provide data supporting both of the hypotheses mentioned above, but there seems to be no fixed pattern regarding the evolutionary trajectories followed by plant species in the QTP. Additionally, no species has been reported to recolonize the QTP from its southern edge (Qu, Lei, Zhang, & Lu, 2010; Yu et al., 2017), which is unexpected given the reports of southern refugia in Europe and North America (Hewitt, 2000), the presence of several south–north valleys connecting the southern edge of the QTP to its interior (Garzione, DeCelles, Hodkinson, Ojha, & Upreti, 2003), and the distributions of certain species that occur on both sides of the Himalayas (Opgenoorth et al., 2010; Qu et al., 2010; Ren et al., 2017).

The southern QTP is a rugged region characterized by high biodiversity and high endemism (Zhang, Ye, & Sun, 2016), and its most prominent topographic feature is the Yarlung Zangbo River valleys between the Himalayas and the Gangdese‐Nyainqentanglha Range. Steep slopes within 10 km of the Yarlung Zangbo River and its tributaries lead to differences in elevation of more than 3,000 m (Wang et al., 2014) and significant altitudinal variation in vegetation, which ranges from dry riparian vegetation up to alpine meadows (Wu & Wu, 1998). Opgenoorth et al. (2010) argued that the topography of the southern QTP and the neighboring Hengduan Mountains is more varied in comparison with that of the northern QTP, and their diverse habitats enabled more species to survive glaciations in situ. In contrast, several species have recolonized the southern QTP from refugia in the southeastern edge of the QTP (Cun & Wang, 2010; Yang et al., 2008; Yu et al., 2017). Thus, differences in the demographic histories of species in the southern QTP may have been caused by their specific biological characteristics such as habitat preferences or dispersal capacity.

To test whether different habitat preferences can lead to different evolutionary trajectories under a shared climatic and geological background, and whether plant species can recolonize the QTP from its southern edge, we focused on *Rhodiola* sect. *Prainia*, a monophyletic taxon consisting of *Rhodiola stapfii,* and *R. prainii*. Currently, these two species have a similar distribution pattern in the southern QTP, but they occur in different habitats.

*R. stapfii* and *R. prainii* are both distributed disjunctively on mountains around the valleys of the Yarlung Zangbo River and the southern edge of the QTP. Moreover, they form a species pair (Zhang, Meng, Allen, Wen, & Rao, 2014), and share many features. The most conspicuous difference between these species is their distinct habitat preferences. *R. prainii* occurs on rock slopes or cliffs, while *R. stapfii* is restricted to alpine meadows. Alpine meadows are common at high elevations in the QTP, while rock slopes are distributed discontinuously along river valleys or suture zones. If the biogeographic histories of *R. stapfii* and *R. prainii* were dominated by shared geographic events and climate change, then a synchronic evolutionary history would be expected. Otherwise, the influence of different habitat preferences on glacial survival and postglacial dispersal should be taken into consideration.

In this study, 19 populations of *R. stapfii* and *R. prainii* were sampled across their distribution areas, including both the southern edge and interior of the QTP. Plastid sequences, nuclear internal transcribed spacer (ITS) sequences, and nuclear microsatellites (simple sequence repeats, SSRs) were used to elucidate the phylogenetic relationships, phylogeographic patterns, and demographic histories of the studied species. In addition, their historical distributions and migration routes were reconstructed using species distribution models (SDMs). Furthermore, we assessed whether these two closely related species behaved differently under the shared influence of Quaternary climate change, and whether they recolonized the interior of the QTP from its southern edge.

## 2 MATERIALS AND METHODS

### 2.1 Sampling and DNA extraction

**Figure 1:**
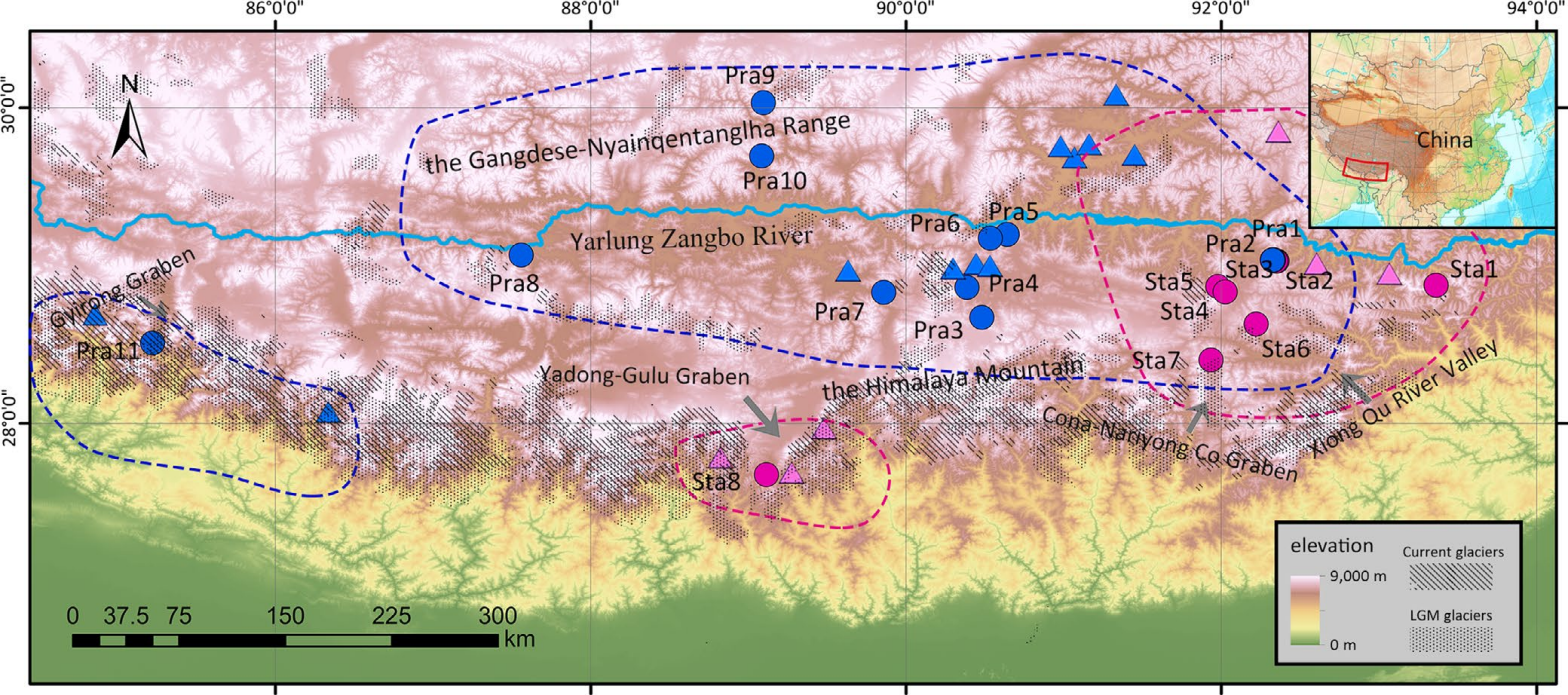
Distribution range (the dashed lines) and records (the circles) of *Rhodiola stapfii* (red) and *R. prainii* (blue). Filled circles indicate sampling sites of this study and open circles represent records collected from online data sets. The extent of ice sheets is reconstructed based on Shi (2008)

**Figure 2:**
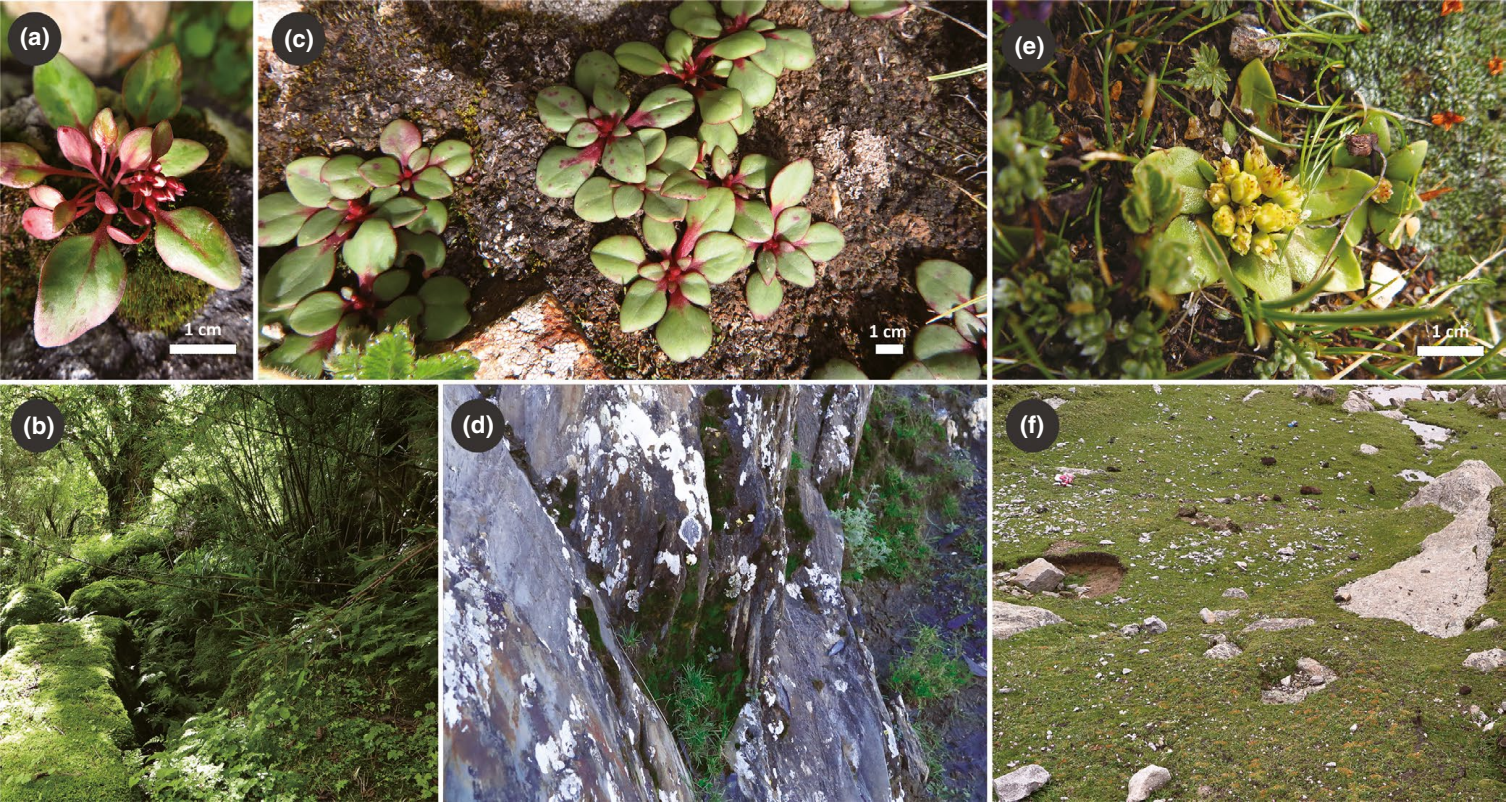
Morphology and habitats of *Rhodiola prainii* and *Rhodiola stapfii*. (a) Morphology of *R. prainii* population Pra11; (b) Pra11 occurs on wet rocks in forests of the Gyirong Graben in the Himalayas; (c) morphology of *R. prainii* populations Pra1 through Pra10; (d) Pra1 through Pra10 occur on rock slops or crevices on open ground; (e) morphology of *R. stapfii*; (f) *R. stapfii* occurs in alpine meadows

Samples were collected from the mountains and shallow valleys of the southern edge and interior of the QTP (Figure 1). In this study, the southern edge of the QTP refers to the southward‐flowing watersheds at the southern slope of the Himalayas. Leaves were collected from 240 individuals from 19 populations of *R. prainii* and *R. stapfii* (eight *R. stapfii* populations and eleven *R. prainii* populations) in the field throughout their distributions and dried in silica gel (see Table S1). Although a detailed field investigation was conducted at the southern edge of the QTP, only one population was sampled in this region for *R. prainii* (Pra11) and *R. stapfii* (Sta8), respectively. Plants from population Pra11 occurred on wet rocks under dense forests in Gyirong Graben, while the remaining populations (Pra1 through Pra10) were found on rock slopes in open habitats in the inner part of the southern QTP (Figure 2). Samples were also collected from closely related species as outgroups (Table S1). Genomic DNA was extracted following Zhang et al. (2014).

### 2.2 Genetic marker design and detection

Microsatellite markers and informative plastid DNA markers were developed from high‐throughput sequencing data as follows (Table S2). Five plastid genomes (three *R. prainii* and two *R. stapfii*) were acquired following Dong, Xu, Cheng, Lin, and Zhou (2013). Five pairs of plastid primers were designed to cover the most informative regions on the plastid genomes according to the following criteria: (a) the PCR product should be less than 500 bp in length; (b) primers should be located in coding regions; (c) the fragment should cover as much variation as possible; (d) SSR or poly A/T regions should be avoided (Table S4). With regard to traditional plastid markers, only *trn*H‐*psb*A (Sang, Crawford, & Stuessy, 1997) was used because it contains abundant variation in *R. stapfii* and *R. stapfii* (Table S3). Thus, six plastid markers in total were used in this study. As to nuclear markers, nuclear reads were assembled de novo into short contigs, from which SSR loci were detected and their primers designed. Next, 165 pairs of SSR primers were chosen randomly and screened for suitable stability and polymorphisms in a sample consisting of three individuals from each population. Finally, 13 robust and highly informative SSR loci were selected for genotyping of all individuals of both species (Table S5). Traditional marker ITS sequences were also used (Mayuzumi & Ohba, 2004). PCR was performed following Zhang et al. (2014). SSRs were amplified and detected following Schuelke (2000).

### 2.3 Phylogenetic analysis

To confirm the relatedness of the two studied species, a phylogenetic analysis including several closely related species was carried out. DNA sequences were aligned with muscle 3.8.31 (Edgar, 2004) and checked manually. ITS ribotypes and concatenated plastid haplotypes were generated with dnasp 5.10.01 (Librado & Rozas, 2009). Haplotype networks of concatenated plastid sequences were drawn using the TCS method implemented in popart 1.7 (Leigh & Bryant, 2015). Phylogenetic analyses were conducted within a Bayesian framework using beast 1.8.4 (Drummond & Rambaut, 2007). The ITS tree was dated by setting the divergence time between *Rhodiola* and *Phedimus* in beast to 21.02 Ma with a standard deviation of 6.5 Ma (Zhang et al., 2014). Under the Bayesian information criterion (Schwarz, 1978), jmodeltest 2.1.7 (Darriba, Taboada, Doallo, & Posada, 2012) selected K2 + G (Kimura, 1980) as the best‐fit substitution model for the ITS sequences, whereas TN93 (Tamura & Nei, 1993) was selected for the concatenated plastid sequences. Convergence was checked using TRACER 1.7 (Rambaut, Drummond, Xie, Baele, & Suchard, 2018). Phylogenetic analyses were also conducted using the maximum parsimony (MP) method implemented in paup 4 (Swofford, 2003) and the Bayesian approach in mrbayes 3.2.6 (Ronquist et al., 2012; Figure S1–S4).

### 2.4 Population genetic structure

For SSR data, diversity indices (*H*_e_, *H*_o_) were calculated in arlequin 3.5.2.2 (Excoffier & Lischer, 2010). The genetic structure was inferred based on SSR loci using structure 2.3.4 (Pritchard, Stephens, & Donnelly, 2000) assuming an admixture model. The burn‐in was set to 20,000, and 50,000 generations were run after it. The *K*‐value was then set to range from 1 to 12, and the computation was repeated 10 times for each *K*‐value. Convergence was reached if alpha value arrived at a plateau in each run and 10 different runs with the same *K*‐value resulted in similar results (Porras‐Hurtado et al., 2013). The *ΔK* criterion implemented in structure harvester v0.6.94 (Earl & vonHoldt, 2012) could not provide a clear best *K*‐value in this study (Figure S5; Table S5), possibly because the *ΔK* criterion is insensitive to the second‐level genetic structure under complex schemes (Evanno, Regnaut, & Goudet, 2005). A clear population structure emerged when *K* = 3 or 4. clumpp 1.1.2 (Jakobsson & Rosenberg, 2007) and distruct 1.1 (Rosenberg, 2004) were used to summarize and visualize the structure results.

In addition, the genetic differentiation among populations within each evolutionary lineage was estimated using analysis of molecular variance (AMOVA) in arlequin.

### 2.5 Recent population dynamics

**Figure 3:**
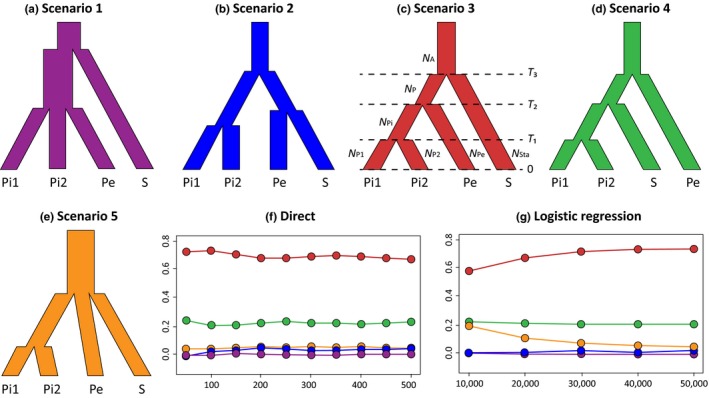
(a–e) The five scenarios tested in the approximate Bayesian analysis and scenario comparison. Pi1: populations Pra1‐Pra2; Pi2: Populations Pra3‐Pra10; Pe: Pra11; S: Populations Sta1‐Sta8. (f, g) Comparison between the five scenarios. Simulations closest to observation are on the *x*‐axes and percentages of each scenario are on the *y*‐axes

**Figure 4:**
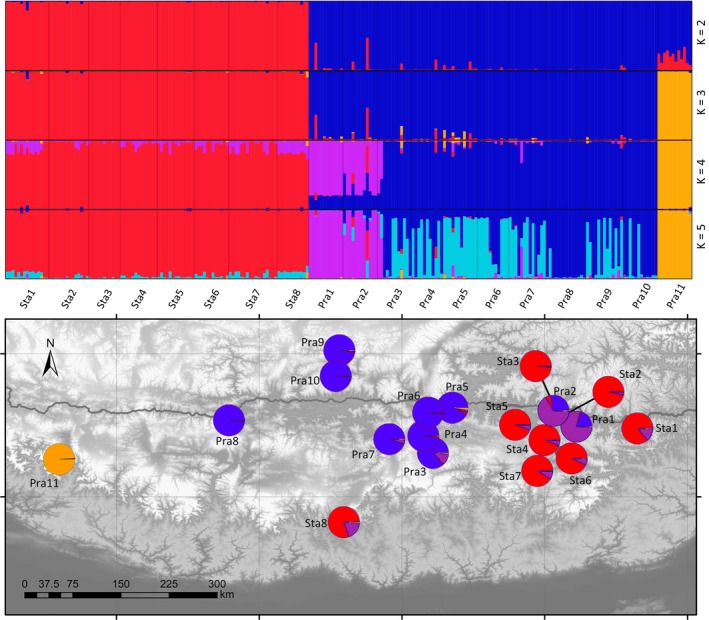
Inferred population structure of *Rhodiola* sect. *Prainia* based SSRs and its geographic distribution (*K* = 4)

Neutrality tests on DNA sequences and bottleneck analysis on SSR data were used to test for possible postglacial expansions of the two studied species. dnasp was used to calculate Tajima's *D*, Fu and Li's D*, and Fu and Li's F* based on ITS sequences to identify signatures of demographic expansions (Librado & Rozas, 2009), and 10,000 coalescent simulations were run to estimate the *p*‐value of each index. Changes in population size for different groups were calculated using a two‐phase model (TPM) with 95% single‐step mutations and a variance among multiple steps of 12 in bottleneck 1.2 using the SSR data (Piry, Luikart, & Cornuet, 1999). A sign test, a standardized differences test, and Wilcoxon's signed rank test were performed in bottleneck to determine whether there was a heterozygosity deficit. Frequency of private SSR alleles (alleles detected in only one population of Sect. *Prainia*; Slatkin & Takahata, 1985) in each population were calculated in R (for scripts see Appendix S2 files). Ten individuals were randomly selected 100 times for the target population to correct the uneven population size, and the number of private alleles was averaged across replicates. The approximate Bayesian computation (ABC) approach implemented in diyabc 2.1.0 (Cornuet et al., 2014) was used to compare five possible evolutionary scenarios for populations of *R. stapfii* and *R. prainii* (Figure 3). All populations were divided into four groups before ABC according to the structure results at *k* = 4 (Figure 4). Mean number of alleles and mean genic diversity of each group are summarized in diyabc. Finally, 5,000,000 simulated data sets were generated, and the 10,000 data sets that were closest to the observed data set were used to estimate each parameter.

### 2.6 Environmental factor analysis and niche modeling

**Figure 5:**
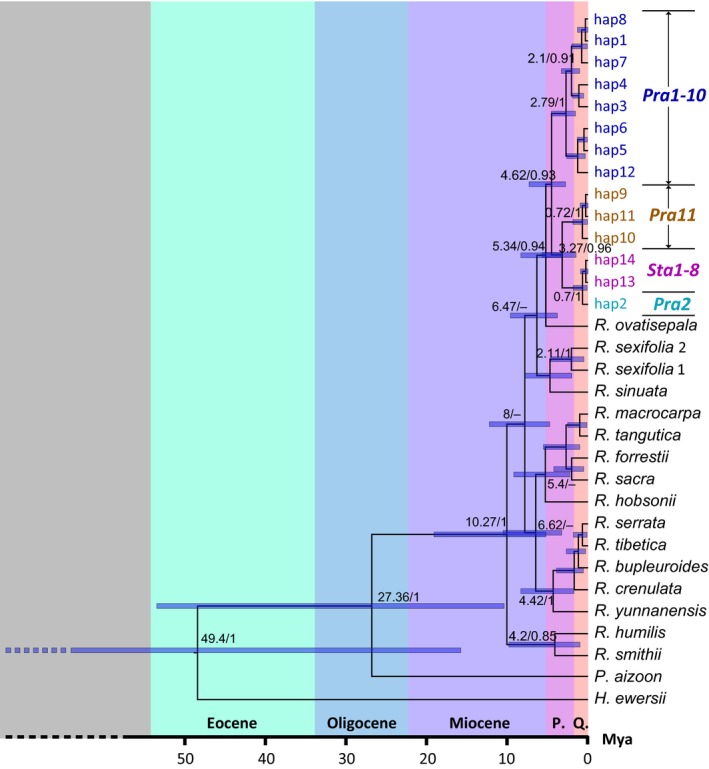
Dated Bayesian tree of the studies species based on nuclear ribosomal DNA internal transcribed spacer (ITS) sequences. Critical nodes are labeled with “node age (million years, Ma)/posterior probabilities (PP).” PP values smaller than 0.7 are not shown

To understand how the distributions of *R. prainii* and *R. stapfii* changed after the LGM, they were modeled under three climatic scenarios from the CCSM4 model (current climate at 30 arc‐seconds resolution, mid‐Holocene 6,000 years ago and the LGM 21,000 years ago at 2.5 arc‐minutes resolution) with maxent 3.3.3k (Phillips & Dudík, 2008). Nineteen ecological factors were acquired from worldclim 1.4 (available at: www.worldclim.org; Hijmans, Cameron, Parra, Jones, & Jarvis, 2005). Current environmental data for 19 sampling sites as well as 30 occurrence records from our field investigation and online data sets (Table S7) were used in this study. To avoid collinearity, Pearson pairwise correlation analysis of environmental factors was conducted, and one factor was eliminated in each pair with a correlation value higher than 0.8 (Table S8; Ren et al., 2017). The selected factors were also used to describe the niche of each population with principal component analysis (PCA) using the “princomp” function in r 3.3.1 (R Core Team, 2016). SDMs were built under the maximum entropy method implemented in maxent with the settings reported by Papeş and Gaubert (2007). Records of the occurrence of *R. prainii* at the southern edge of the QTP were not included in the SDM analysis because they form a lineage different from other *R. prainii* populations in the interior QTP (Figure 5) and occur in different environments. Selected rasters of environmental factors were cropped to span from 26°N to 33°N and from 78°E to 100°E before modeling.

## 3 RESULTS

### 3.1 Microsatellite marker design and selection

According to analyses of high‐throughput sequencing data from five individuals of the *Rhodiola* sect. *Prainia* species (two *R. stapfii* and three *R. prainii*), an average of 15,499,029 clean reads were acquired for each individual, from which 2,514 pairs of nuclear SSR primers were designed. Finally, 13 robust and highly informative SSR loci were used for the population genetic analysis of the Sect. *Prainia* species: *R. stapfii* and *R. prainii* (Table S5).

### 3.2 Phylogenetic and haplotype analysis

**Figure 6:**
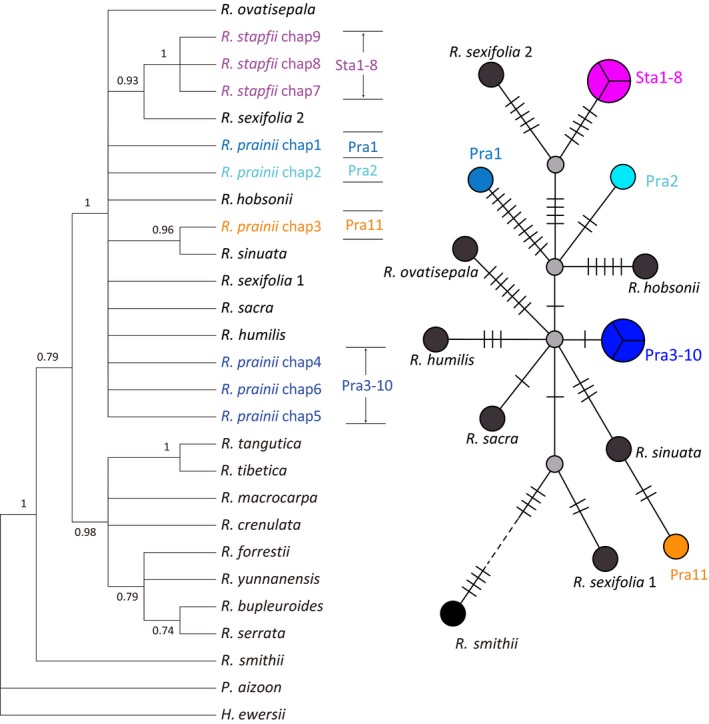
Bayesian tree and haplotype network of the concatenated plastid sequences of the studied species. Critical nodes on the phylogenetic tree are labeled with posterior probabilities. PP values smaller than 0.7 are not shown. Haplotype network was built using the TCS method implemented in popart

The final set of DNA sequences consisted of 570 bp of the ITS and 2081 bp of concatenated plastid sequences. Fourteen ITS ribotypes and nine plastid haplotypes were detected across *R. stapfii* and *R. prainii* (Table 1). The phylogenetic trees for the ITS and concatenated plastid sequences were incongruent (Figures 5, 6). The ITS tree showed that all populations of *Rhodiola* sect. *Prainia* constituted a monophyletic clade (posterior probabilities [PP] = 0.93) with 14 ITS ribotypes separated from *R. ovatisepala* (Figure 5). The ITS ribotypes of *Rhodiola* sect. *Prainia* were distributed in three well‐supported subclades: (a) all ribotypes of *R. prainii* populations in the interior QTP (Pra1 through Pra10, PP = 1) except for one (hap2) from population Pra2; (b) the population Pra11 ribotypes (PP = 1); (c) ribotypes from populations of *R. stapfii* and one rare ribotype (hap2) from population Pra2 (PP = 1; Figure 5). With respect to plastid sequences, there were nine haplotypes in *Rhodiola* sect. *Prainia*: six for *R. prainii* and three for *R. stapfii* (Table 1). The phylogenetic tree of concatenated plastid sequences showed a pattern of polytomy with haplotypes from *R. stapfii*, population Pra1, population Pra2, populations Pra3 through Pra10, and population Pra11, as well as those of other species mixed together (Figure 6). The Bayesian tree constructed using mrbayes and the MP tree constructed using paup also support these results (Figures S1–S4).

**Table 1:** Internal transcribed spacer (ITS) ribotypes, plastid haplotypes and bottleneck analysis of *R. prainii* and *R. stapfii* populations

Pop ID	Individual numbers	ITS ribotype (numbers)	Plastid haplotype (numbers)	SSR						
				*H*_e_	*H*_o_	*N*_e_	*P*_signtest_	*P*_stdv_	*P*_wilcoxon_	*Freq*_(private allele)_
Pra1	12	Hap1 (24)	Chap1 (12)	0.467	0.392	23.38	0.076	0.040*	0.322	0
Pra2	12	Hap1 (14) Hap2 (1) Hap3 (3) Hap4 (3)	Chap2 (12)	0.411	0.315	22.77	0.366	0.152	0.414	0.046
Pra3	12	Hap1 (17) Hap6 (2) Hap8 (2) Hap12 (3)	Chap4 (11) Chap5 (1)	0.408	0.303	21.69	0.304	0.120	0.557	0.002
Pra4	11	Hap1 (15) Hap6 (4) Hap8 (2) Hap12 (1)	Chap4 (11)	0.446	0.332	20.15	0.264	0.359	0.625	0.004
Pra5	12	Hap1 (16) Hap6 (8)	Chap6 (12)	0.457	0.404	23.54	0.247	0.104	0.432	0.003
Pra6	11	Hap1 (22)	Chap4 (9) Chap5 (2)	0.468	0.343	21.85	0.399	0.273	0.359	0
Pra7	13	Hap1 (17) Hap5 (2) Hap6 (4) Hap7 (3)	Chap5 (12) Chap6 (1)	0.423	0.314	24.77	0.182	0.095	0.206	0.006
Pra8	14	Hap1 (25) Hap8 (3)	Chap4 (14)	0.314	0.317	27.69	0.585	0.009**	0.570	0.011
Pra9	13	Hap1 (24) Hap8 (2)	Chap4 (13)	0.431	0.343	25.69	0.071	0.018*	0.193	0.004
Pra10	12	Hap1 (24)	Chap4 (11) Chap5 (1)	0.355	0.292	23.85	0.549	0.025*	0.232	0.012
Pra1−10	122	Hap1‐Hap8, Hap12	Chap1, 2, 4, 5	0.409	0.264	235.38	0.000**	0.000**	0.000**	‐
Pra11	12	Hap9 (20) Hap10 (1) Hap11 (3)	Chap3 (12)	0.503	0.427	23.69	0.565	0.227	0.547	0.215
Sta1	15	Hap13 (30)	Chap7 (15)	0.355	0.340	29.69	0.049*	0.001**	0.020*	0.005
Sta2	14	Hap13 (28)	Chap7 (14)	0.335	0.338	27.69	0.422	0.440	0.938	0
Sta3	11	Hap13 (22)	Chap7 (11)	0.397	0.387	21.54	0.576	0.419	0.844	0
Sta4	13	Hap13 (26)	Chap7 (11) Chap8 (1) Chap9 (1)	0.36	0.39	25.38	0.461	0.432	1.000	0
Sta5	13	Hap13 (26)	Chap7 (12) Chap8 (1)	0.407	0.349	25.85	0.229	0.127	0.688	0
Sta6	12	Hap13 (24)	Chap7 (12)	0.291	0.263	23.69	0.203	0.039*	0.109	0
Sta7	17	Hap13 (34)	Chap7 (15) Chap8 (1) Chap9 (1)	0.348	0.338	33.08	0.446	0.224	0.469	0
Sta8	11	Hap14 (22)	Chap7 (11)	0.303	0.251	21.23	0.061	0.009**	0.031*	0.006
Sta1‐8	106	Hap13, 14	Chap7, 8, 9	0.258	0.223	208.15	0.001**	0.000**	0.001**	‐
Total	240	Hap1‐14	Chap1‐9	0.576	0.223	467.23	0.001**	0.000**	0.003**	‐

Note

*H*_e_ and *H*_o_ were calculated in arlequin. bottleneck analysis of each population and separate pooled populations within each of the three genetic lineages was performed using the two‐phase model with 95% single‐step mutations and a variance of 12 among multiple steps. *p < 0.05; **p < 0.01. *N*_e_: Current effective population size generated by bottleneck. Freq_(private allele)_: frequency of private alleles.

### 3.3 Population genetic structure

When *K* = 3, structure analysis based on SSRs designated all individuals of *Rhodiola* sect. *Prainia* into three genetically distinct clusters, which corresponded to all populations (Sta1 through Sta8) of *R. stapfii*, *R. prainii* population Pra11, and *R. prainii* populations Pra1 through Pra10 (Figure 4). These results are in accordance with those of the phylogenetic analysis based on ITS sequences (Figure 5). When *K* = 4, populations Pra1 through Pra10 were further divided into two subclusters, which occurred in the middle and eastern part of the southern QTP, respectively (Figure 4). AMOVA showed that the genetic differentiation is low among populations within each lineage of Sect. *Prainia* (Table 2), which is consistent with the low level of differentiation found within each cluster by the structure analysis at *K* = 3 (Figure 4).

**Table 2:** Analysis of molecular variance (AMOVA) for the SSR data

Source of variation		*d. f*.	Sum of squares	Variance components	Percentage of variation (%)
*R. prainii* (Pra1‐10)	Among populations within Pra1‐10	9	104.400	0.4181	22.9
	Within populations of Pra1‐10	234	328.404	1.4034	77.1
*R. stapfii*	Among populations within *R. stapfii*	7	21.158	0.0853	10.0
	Within populations of *R. stapfii*	204	156.724	0.7683	90.0
Pra11	Within populations of Pra11	23	46.333	2.0284	‐

Note

Analysis was performed independently within each of the three genetic lineages of *Rhodiola* sect. *Prainia*.

### 3.4 Recent population dynamics

Tajima's *D*, Fu & Li's D* and Fu & Li's F* tests on ITS sequences within each of the three genetic lineages did not provide strong evidence for recent expansions/bottlenecks (Table 3). bottleneck analysis based on the SSRs of separate pooled populations within each of the three genetic lineages indicated that all populations of *R. stapfii* and interior populations of *R. prainii* had excess heterozygote deficiency at the population level, which was suggestive of recent bottleneck events (Table 1). Further tests of each population showed that only populations Sta1 and Sta8 underwent recent bottleneck events (Table 1). These findings seem paradoxical, but there were no contradictions because “recent” in bottleneck was defined as within approximately the past 2*N_e_*–4*N_e_* generations (Piry et al., 1999), while the results of the pooled population analyses reflected their common demographic history, which was much longer than that of a single population (Table 1). ABC analysis identified scenario 3 as the best evolutionary model for *Rhodiola* sect. *Prainia*, which suggests that populations of *R. stapfii* diverged first, after which populations of *R. prainii* split into two lineages on both sides of the Himalayas (Figure 3). Considering that the generation time of the studied species is estimated to be approximately five years based on our field observation and previous studies (Galambosi, 2014; Wu, Shang, Dai, & Yan, 2001), the split time between populations Pra1 to Pra2 and Pra3 to Pra10 (*T*_1_) was estimated at ~511 [305–729] generations (Table 4), which should be after the LGM. The split time between the interior plateau (Pra1 through Pra10) and the southern edge populations (Pra11) of *R. prainii* (*T*_2_) was estimated at ~9,320 [3,980–27,400] generations, (Table 4), which would be earlier than the LGM.

**Table 3:** Population dynamic analysis based on the ITS sequences within each of the three genetic lineages of *Rhodiola* sect. *Prainia*

	Tajima's *D*	*P*_(sim<obs)_	Fu & Li's D*	*P*_(sim<obs)_	Fu & Li's F*	*P*_(sim<obs)_
*R. stapfii*	−0.015	0.556	−2.70537	0.067	−2.203	0.060
*R. prainii* (Pra1‐Pra10)	−0.322	0.422	1.741	0.998^*^	1.178	0.922
*R. prainii* (Pra11)	1.724	0.947	1.044	0.655	1.400	0.949

Note

*p*‐Values were generated based on 10,000 coalescent simulations in dnasp.

**Table 4:** Parameters estimated from scenario 3 in the approximate Bayesian computation analysis based on SSRs of *Rhodiola* sect. *Prainia*

Parameter	Mean	Median	Q_0.25_	Q_0.75_
*N*_Sta_	388	295	160	512
*N*_P1_	644	162	74.7	371
*N*_P2_	5,200	2,470	1,190	4,970
*N*_Pe_	221	151	74.1	291
*N*_Pi_	531	296	107	716
*N*_P_	4,260	245	30.7	2,370
*N*_A_	3,920	326	41.7	2,930
*T*_1_	518	511	305	729
*T*_2_	32,400	9,320	3,980	27,400
*T*_3_	36,200,000	30,100,000	12,700,000	55,900,000

Notes

*N*_p1_, *N*_p2_, *N*_pe_, *N*_sta_, *N*_pi_, *N*_p_ and *N*_A_ stand for effective population size of populations Pra1‐Pra2, populations Pra3‐Pra10, Pra11 populations Sta1‐Sta8, ancestral population of populations Pra1‐Pra10, ancestral population of populations Pra1‐Pra10 and ancestral population of *R. stapfii* and *R. prainii*. *T*_1_, *T*_2,_ and *T*_3_ stands for split time in generations between Pra1‐Pra2 and Pra3‐Pra10, Pra1‐10 and Pra11, and between *R. stapfii* and *R. prainii*.

### 3.5 Environmental factor analysis and SDMs

**Figure 7:**
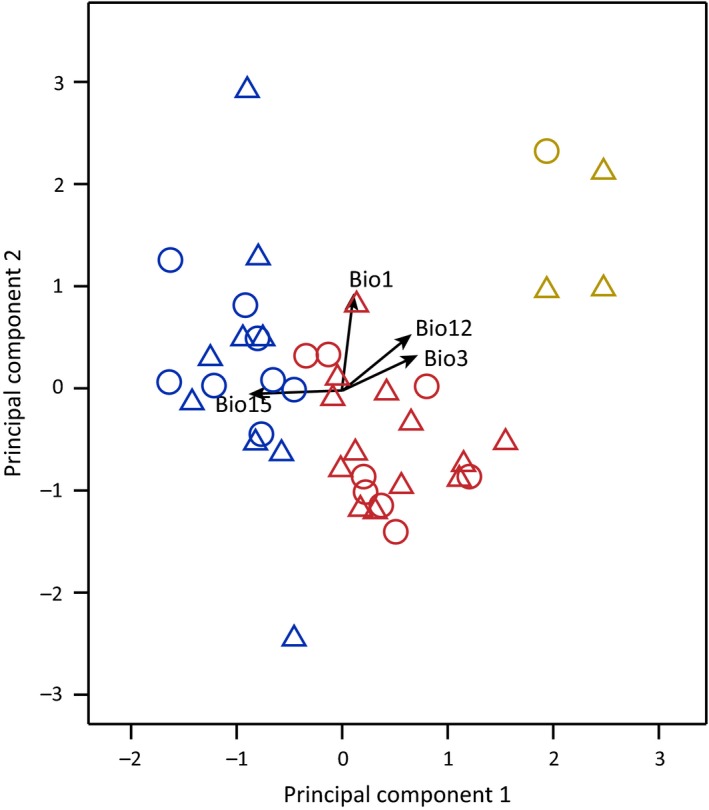
Principal component analysis (PCA) of the environmental factors at the occurrence of *Rhodiola* sect. *Prainia*. The solid lines indicate the niches of the occurrence sites of *R. stapfii* (red) and *R. prainii* from the interior of the QTP (dark blue) and *R. prainii* from the southern slope of the QTP (orange). The solid border circles indicate the populations sampled in this study while the triangles indicate occurrence data from other sources (Talbe S7). Bio 1: annual mean temperature; Bio 3: isothermality; Bio 12: annual precipitation; Bio 15: precipitation seasonality

**Figure 8:**
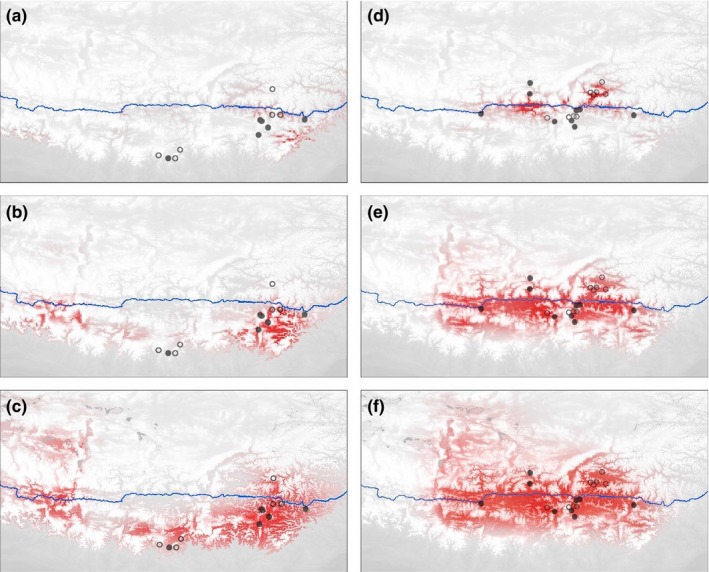
Predicted distributions of *R. stapfii* and interior populations of *R. prainii*. The color density indicates the probability of occurrence. The predicted distributions of *R. stapfii* during the Last Glacial Maximum (a), mid‐Holocene (b), and current period (c) are shown on the left. The predicted distributions of interior populations of *R. prainii* during the Last Glacial Maximum (d), mid‐Holocene, (e) and current period (f) are shown on the right. Circles indicate population sites used in species distribution modeling (filled circles indicate sampling sites of this study and open circles represent records collected from online data sets)

To reduce multicollinearity, one factor of each pair of environmental factors was eliminated when the correlation coefficient of the pair was >0.8. Thus, only four factors were left: annual mean temperature, isothermality, annual precipitation, and precipitation seasonality (Table S8). PCA of the four environmental factors at all known locations of *Rhodiola* sect. *Prainia* showed that the first two principal components accounted for 79.6% of the total variance (Table 5). Compared to populations Sta1 through Sta8, populations Pra1 through Pra10 resided in places with a higher second component (PC2), while Pra11 resided in places with the highest first component (PC1) and lowest PC2 (Figure 7). These results were consistent with our field observation that populations Sta1 through Sta8, populations Pra1 through Pra10, and population Pra11 occurred in different habitats (Figure 2). The predicted distributions of *R. stapfii* and *R. prainii* (excluding population Pra11 of *R. prainii* from the southern edge of the QTP) during the LGM showed that *R. prainii* retreated to valleys of the middle reaches of the Yarlung Zangbo River (Figure 8d), whereas *R. stapfii* retreated to Xiong Qu Valley at the southeastern edge of the QTP or valleys in the lower reaches of the Yarlung Zangbo River. After the LGM, *R. prainii* expanded across valleys from its refugium, which is located in the center of its current distribution, while *R. stapfii* expanded generally westward from its refugium (Figure 8). The predicted current distribution of *R. prainii* covers the valleys of the middle reaches of the Yarlung Zangbo River and its tributaries (Figure 8f), whereas that of *R. stapfii* covers the eastern and western parts of the southern QTP and the area along the middle Himalayas (Figure 8c).

**Table 5:** Loadings from the PCA of the niche of each population of *R. prainii* and *R. stapfii*. Bio 1: annual mean temperature; Bio 3: isothermality; Bio 12: annual precipitation; Bio 15: precipitation seasonality

	Cumulative variance	Bio 1	Bio3	Bio 12	Bio 15
PC1	0.469	0.142	0.733	0.674	−0.930
PC2	0.796	0.962	0.334	0.519	−0.007

## 4 DISCUSSION

### 4.1 Phylogenetic relationship among lineages of Rhodiola sect. Prainia

The incongruence of the topologies of the gene trees constructed from the ITS and plastid sequences may have been the result of inadequate phylogenetic information, inaccurate species limits, incomplete lineage sorting, unrecognized paralogy, or hybridization (Funk & Omland, 2003). The high posterior probabilities of the three major clades on the *Rhodiola* sect. *Prainia* ITS tree argued against inadequate phylogenetic information and inaccurate species limits. The effects of unrecognized paralogy were ruled out due to concerted ITS evolution. The ITS ribotypes of *Rhodiola* sect. *Prainia* formed a monophyly with three well‐supported clades (Figure 5), while the plastid haplotypes of this section were mixed with those of other species (Figure 6). This finding could not be explained by incomplete lineage sorting because species of *Rhodiola* sect. *Prainia* are generally distributed in small patches and show predominantly sexual reproduction, which should lead to fast sorting of ancient haplotypes. The low intra‐population differences in plastid sequences also support fast sorting in populations of *Rhodiola* sect. *Prainia* (Figure 6). Plastid haplotype chap3 of *R. prainii* population Pra11 clustered together with that of *R. sinuata* occurring in the same locality (Gyirong Graben), which suggests that there might be a recent introgression between these two species. Therefore, plastid capture through strong introgression between co‐distributed species would be the most likely explanation.

The ABC analysis of SSRs supported a branching order for the three lineages that was different from that suggested by the ITS tree. The most supported model in the ABC analysis (Scenario 3) suggested that *R. stapfii* diverged first from the ancestor of all populations of *R. prainii*, which is consistent with the fact that *R. prainii* at the southern edge of the QTP (Pra11) is morphologically more similar to *R. prainii* in the interior QTP than to *R. stapfii* (Figure 2). The discrepancy between the results obtained from SSRs and ITS sequences could be the result of incomplete sorting of ancestral polymorphisms or stochastic introgression of ITS or SSR loci. These findings suggest that *Rhodiola* sect. *Prainia* consists of three genetic lineages, and population Pra11 can be treated as an independent phylogenetic species according to the species concepts of Nixon and Wheeler (1990).

### 4.2 Evolutionary histories of* R. prainii* and* R. stapfii*

The lack of genetic structure within *R. stapfii* suggests that current populations could be derived from only one refugium after the LGM (Table 2; Figure 3; Hewitt, 2000). The same conclusion could be drawn for *R. prainii* populations Pra1 through Pra10. The SDM results (Figure 8d) suggest that a valley in the middle reaches of the Yarlung Zangbo River may have been the glacial refugium for *R. prainii* (excluding population Pra11) during the LGM. After the LGM, *R. prainii* expanded out from this glacial refugium onto the surrounding mountains (Figure 8e, f) in a manner corresponding with the in situ survival hypothesis. In contrast, the most likely refugium of *R. stapfii* was located in the Xiong Qu Valley (an eastward valley upstream of the Subansiri River) (Figure 8a). After the LGM, *R. stapfii* spread out from the Xiong Qu Valley and migrated westwards to the location of population Sta9 at the southern edge of the QTP to form its current distribution pattern. Therefore, the survival history of *R. stapfii* during the LGM corresponded with the *tabula rasa* hypothesis. The Himalayas served as westward migration corridors rather than geographic barrier for *R. stapfii*, which is a recurrent pattern exhibited by other alpine species of this region (Wallis, Waters, Upton, & Craw, 2016; Yu et al., 2017). In addition, the valleys of the Yarlung Zangbo River were also potential refugia for *R. stapfii* (Figure 8a), in which case the *tabula rasa* hypothesis would be inaccurate. Evolutionary scenarios in mountainous regions could be far more complex than the simple dichotomy of the in situ survival versus *tabula rasa* hypotheses (Sersic et al., 2011; Stehlik, 2003), and a mixed survival scenario could not be ruled out in the case of *R. stapfii*.

ABC analysis showed that the establishment of the three lineages of *Rhodiola* sect. *Prainia* was earlier than the LGM (Table 4; Figure 3), and this finding was also supported by the dated phylogenetic tree of the *Rhodiola* sect. *Prainia* ITS sequences (Figure 5). These results suggest that the three lineages survived several earlier glaciations that were more extensive than the LGM (Lehmkuhl & Owen, 2005). Therefore, due to its limited distribution and strong contractions during the LGM, the evolutionary history of Sect. *Prainia* before the LGM cannot be conjectured based on the results of this study.

### 4.3 Valley refugia

Both *R. prainii* and *R. stapfii* survived glaciations in deep river valleys. During glaciations, the valleys of the Yarlung Zangbo River were mostly unglaciated (Owen & Dortch, 2014) and provided glacial refugia for many local species at a wide range of elevations because of their significant climatic buffering capacity (Frenzel, Bräuning, & Adamczyk, 2003; Liang, He, Jia, Sun, & Chen, 2017; Opgenoorth et al., 2010; Zhang, Comes, & Sun, 2011). The significant role of valleys as glacial refugia is also documented in Patagonia (Sersic et al., 2011), indicating that “valley refugia” might be a common pattern in mountainous regions.

However, deep valleys are not sufficient as glacial refugia during the LGM. The current distribution of *R. stapfii* covers several deep river valleys (Figure 1), but the species had only one refugium during the LGM. The SDM results also suggest that southward valleys along the southern edge of the QTP were unsuitable for *R. stapfii* and *R. prainii* during the LGM (Figure 8).

### 4.4 Lack of recolonization of the interior from the southern edge of the QTP

In some cases, southward valleys at the southern edge of the QTP could serve as glacial refugia, but they do not act as sources of interglacial recolonization (Ren et al., 2017; Opgenoorth et al., 2010; Yu et al., 2017). Under the dominating influence of the Indian monsoon, eastward or southeastward Himalayan valleys showed significant differences in precipitation patterns along elevation gradients in comparison with those of southward valleys (Bookhagen & Burbank, 2010). As a consequence, lineages occurring at the southern edge of the QTP have diverged genetically and ecologically from interior populations for millions of years, which has left them incapable of adapting to the current environments in the interior part of the QTP, as observed for population Pra11 of *R. prainii* (Figures 2, 7).

Another possible explanation could be that recolonization from the southern edge of the QTP was impeded by populations that survived in situ in the interior QTP. Genetic discrepancies resulting from isolated glacial refugia could be maintained even under postglacial contact and introgression (Hewitt, 1996; Wallis et al., 2016). If a large number of populations survived in situ in the interior QTP, migration from the southern edge would lead to a genetic signature of introgression instead of a genetic pattern of recolonization from the southern edge. Such introgression was not found in *Rhodiola* sect. *Prainia*, but it has been documented in the *Juniperus tibetica* complex (Opgenoorth et al., 2010), suggesting that preexisting populations in the interior QTP could block recolonization from the southern edge in some cases. This situation also exists in the southeastern QTP, where some cold‐adapted species recolonized the Himalayas from the Hengduan Mountains across biogeographic barriers, but other cold‐adapted species survived in situ (Cun & Wang, 2010; Li et al., 2013, 2011; Liu et al., 2013; Opgenoorth et al., 2010; Yu et al., 2015).

### 4.5 Migration barriers and corridors

The differences in the postglacial dynamics of *R. stapfii* and *R. prainii* may be the result of differences in habitat preferences. *R. stapfii* occurs on alpine meadows, which have expanded and replaced steppes several times during the mid‐late Holocene in the central QTP (Shen et al., 2008). It is possible that *R. stapfii* expanded following the expansion of alpine meadows and reached the current location of population Sta8. Recent long‐distance dispersal could be an alternative explanation for the distribution of *R. stapfii*; the species has small seeds (0.5–0.8 mm) and occupies open treeless habitats. However, considering that *R. prainii* and *R. stapfii* have similar seeds, long‐distance dispersal may not account for the different migration patterns of *R. stapfii* and *R. prainii* in the interior QTP.

Previous studies on the demographic history of species in the southern QTP identified two common westward migration routes: (a) along the Himalayas and (b) along the Yarlung Zangbo River valleys (Qiu et al., 2011; Yu et al., 2017). In this study, *R. stapfii* migrated along the Himalayas, whereas *R. prainii* expanded in both directions along the valleys of the Yarlung Zangbo River, indicating that the migratory pattern of a species is influenced by its habitat preference, as manifested by characteristics such as habitat preferences, in addition to the locations of glacial refugia.

Although the SDMs predicted that there were suitable habitats for *R. stapfii* in the southwestern Himalayas (Figure 8c) and alpine meadows are widespread in this region (Wang et al., 2016), no *R. stapfii* have been described there. One explanation for this lack of *R. stapfii* could be that the topography of the southern QTP hindered it from reaching the western Himalayas. However, this argument is weakened by the potential shortcomings of field investigations and SDM analysis, and possible dynamics between each static SDMs may be ignored.

### 4.6 Population size dynamics of herbs in the QTP

Although many cold‐adapted species have experienced glacial expansions and interglacial contractions (Stewart et al., 2010), phylogeographic studies of alpine herbs on the southern QTP have not consistently reached the same conclusions (Wang, Abbott, et al., 2009). Four subnival herbs, which occur mostly in rocky environments at elevations between 3,400 and 5,000 m, experienced downslope expansion during the LGM and upslope contraction during postglaciation periods (Luo et al., 2016). Another herb species, *Pedicularis longiflora*, which is distributed in wet meadows and along hill streams at elevations between 2,600 and 5,300 m, experienced contraction during glacial advancement and expansion during glacial retreats (Yang et al., 2008). Asynchronous population size changes were documented in *Primula tibetica*, another herb with four genetic lineages occurring in wet meadows and along streams at elevations between 2,600 and 5,000 m. Two lineages of *P. tibetica* experienced a scenario of “‘expansion‐shrinkage‐expansion’,” while two other lineages experienced a scenario of “‘expansion‐shrinkage”’ (Ren et al., 2017). In this study, *R. stapfii* and *R. prainii* of the inner QTP were sampled from elevations of 4,100–5,000 m. *R. stapfii* from alpine meadows experienced strong contraction during the LGM and expansion afterward, which is similar to the pattern displayed by *Pedicularis longiflora* (Yang et al., 2008). However, *R. prainii* of the inner QTP showed changes similar to those experienced by meadow herbs during glaciations instead of those experienced by species in rocky environments. One possible explanation for this finding could be that *R. prainii* of the inner QTP had a narrow adaptation amplitude that restricted the distribution of this lineage during glaciations. In comparison with other alpine species from rocky environments, *R. prainii* is usually found on rocks with a relatively large amount of moss (per. obs.), which suggests that micro‐habitat preferences might be another explanation for the migratory pattern of this species. These findings suggest that any explanations of differences in changes in population size for herbs on the southern QTP are inevitably influenced by taxon‐specific adaptation characteristics besides cold tolerance.

## 5 CONCLUSION

This study showed that *Rhodiola* sect. *Prainia*, a taxon endemic to the southern QTP, consists of three evolutionary lineages rather than two species as previously described. *R. prainii* distributed at the southern edge of the QTP should be treated as an independent phylogenetic species (Nixon & Wheeler, 1990). The survival strategy of *R. prainii* in the interior part of the QTP during the LGM corresponded with the in situ survival hypothesis, while that of *R. stapfii* probably corresponded most closely with the *tabula rasa* hypothesis. Taken together with previous studies, the findings of this study suggest that southward valleys at the southern edge of the QTP are either not appropriate glacial refugia, or not sources of recolonization to the interior QTP (Cun & Wang, 2010; Opgenoorth et al., 2010; Ren et al., 2017; Yang et al., 2008; Yu et al., 2015).

## CONFLICT OF INTEREST

None declared.

## AUTHORS CONTRIBUTION

G.R. and Z.W. conceived the ideas. Z.W. and S.M. conducted the field research. Z.W. did the laboratory work and analyzed data, supervised by G.R. All authors participated in drafting the manuscript.

## Data Availability

DNA sequences: GenBank accessions MG917775–MG917968.
