# Supplementary material for: Quaternary climate change and habitat preference shaped the genetic differentiation and phylogeography of Rhodiola sect. Prainia in the southern Qinghai–Tibetan Plateau
Source: Ecol Evol. 2019 Jun 30;9(14):8305–19. doi: 10.1002/ece3.5406 (PMC6662313; doi:10.1002/ece3.5406)
Supplement: Supplementary file 1 [file ECE3-9-8305-s001.docx]

# Appendix Files

**Quaternary climate change and habitat preference shaped the genetic differentiation and phylogeography of *Rhodiola* sect. *Prainia* in the southern Qinghai-Tibetan Plateau**

Zi-Meng Wang, Shi-Yong Meng and Guang-Yuan Rao (corresponding author)

**Table S1** Sampling sites of studied species.

| Species | Pop ID | Individual number | longitude | Latitude | Localtion |
| --- | --- | --- | --- | --- | --- |
| *R. prainii* | Pra1 | 12 | 92.330 | 29.038 | Qusum County, Tibet Autonomous Region |
|  | Pra2 | 12 | 92.327 | 29.035 | Qusum County, Tibet Autonomous Region |
|  | Pra3 | 12 | 90.482 | 28.672 | Lhozhag County, Tibet Autonomous Region |
|  | Pra4 | 11 | 90.387 | 28.863 | Nagarze County, Tibet Autonomous Region |
|  | Pra5 | 12 | 90.642 | 29.196 | Quxu County, Tibet Autonomous Region |
|  | Pra6 | 11 | 90.535 | 29.173 | Quxu County, Tibet Autonomous Region |
|  | Pra7 | 13 | 89.858 | 28.831 | Gyantse County, Tibet Autonomous Region |
|  | Pra8 | 14 | 87.559 | 29.066 | Lhatse County, Tibet Autonomous Region |
|  | Pra9 | 13 | 89.096 | 30.032 | Namling County, Tibet Autonomous Region |
|  | Pra10 | 12 | 89.086 | 29.694 | Namling County, Tibet Autonomous Region |
|  | Pra11 | 12 | 85.221 | 28.510 | Gyirong County, Tibet Autonomous Region |
| *R. stapfii* | Sta1 | 15 | 93.362 | 28.876 | Nang County, Tibet Autonomous Region |
|  | Sta2 | 14 | 92.353 | 29.030 | Qusum County, Tibet Autonomous Region |
|  | Sta3 | 11 | 92.323 | 29.034 | Qusum County, Tibet Autonomous Region |
|  | Sta4 | 13 | 91.978 | 28.868 | Nedong District, Tibet Autonomous Region |
|  | Sta5 | 13 | 92.024 | 28.835 | Nedong District, Tibet Autonomous Region |
|  | Sta6 | 12 | 92.221 | 28.630 | Lhunze County, Tibet Autonomous Region |
|  | Sta7 | 17 | 91.935 | 28.402 | Lhunze County, Tibet Autonomous Region |
|  | Sta8 | 11 | 89.115 | 27.676 | Cona County, Tibet Autonomous Region |
| *H. ewersii* | - | 1 | 115.472 | 40.007 | Mentougou District, Beijing City |
| *P. aizoon* | - | 1 | 104.157 | 32.917 | Pingwu County, Sichuan Province |
| *R. bupleuroides* | - | 1 | 101.947 | 30.002 | Kangding City, Sichuan Province |
| *R. crenulata* | - | 1 | 101.905 | 29.988 | Kangding City, Sichuan Province |
| *R. forrestii* | - | 1 | 101.893 | 29.979 | Kangding City, Sichuan Province |
| *R. hobsonii* | Ho | 1 | 91.793 | 29.805 | Maizhokunggar County, Tibet Autonomous Region |
| *R. humilis* | Hu | 1 | 92.240 | 29.702 | Maizhokunggar County, Tibet Autonomous Region |
| *R. macrocarpa* | - | 1 | 104.126 | 32.945 | Pingwu County, Sichuan Province |
| *R. sacra* | Sa | 1 | 91.241 | 29.856 | Linzhou County, Tibet Autonomous Region |
| *R. ovatisepala* | Ov | 1 | 93.116 | 29.964 | Gongbo’gyamda County, Tibet Autonomous Region |
| *R. serrata* | - | 1 | 92.554 | 28.990 | Lhunze County, Tibet Autonomous Region |
| *R. sexifolia* | Se1 | 1 | 92.569 | 29.865 | Qusum County, Tibet Autonomous Region |
| *R. sexifolia* | Se2 | 1 | 90.924 | 28.518 | Lhunze County, Tibet Autonomous Region |
| *R. sinuata* | Si | 1 | 85.364 | 28.358 | Gyirong County, Tibet Autonomous Region |
| *R. smithii* | Sm | 1 | 89.283 | 29.360 | Namling County, Tibet Autonomous Region |
| *R. tangutica* | - | 1 | 97.053 | 32.911 | Yushu Autonomous Prefecture, Qinghai Province |
| *R. tibetica* | - | 1 | 93.129 | 29.966 | Gongbo’gyamda County, Tibet Autonomous Region |
| *R. yunnanensis* | - | 1 | 100.231 | 27.094 | Lijiang City, Yunnan Province |

**Table S2** Statistics of high throughput sequencing

| Sample | Total reads | Chloroplast reads | Proportion of chloroplast reads |
| --- | --- | --- | --- |
| Sta1 | 16643250 | 388100 | 2.33% |
| Sta8 | 12380184 | 493037 | 3.98% |
| Pra1 | 14066182 | 1464786 | 10.41% |
| Pra4 | 20016508 | 608624 | 3.04% |
| Pra10 | 14389020 | 699616 | 4.86% |

**Table S3** Variations of each chloroplast primer pair based on five aligned chloroplast genomes. Poly-A/T indels are not counted.

| Source | Sequence | Aligned Length (bp) | Num. SNPs | Num. indels |
| --- | --- | --- | --- | --- |
| Traditional markers | *matK* | 837 | 0 | 3 |
|  | *trnL-trnF* | 958 | 2 | 0 |
|  | *psbA-trnH* | 314 | 3 | 1 |
|  | *rbcL* | 1332 | 2 | 0 |
|  | *rpS16* | 809 | 2 | 0 |
|  | *trnS-trnG* | 1354 | 4 | 1 |
|  | *atpF* | 931 | 2 | 1 |
|  | *trnC-psbM* | 2037 | 9 | 1 |
|  | *psbM-trnD* | 1063 | 3 | 1 |
|  | *trnC-rpoB* | 1100 | 5 | 2 |
|  | *ycf3* | 1666 | 4 | 1 |
|  | *trnS-rpS4* | 801 | 1 | 1 |
|  | *trnT-trnL* | 428 | 0 | 1 |
|  | *trnL* | 537 | 1 | 0 |
|  | *trnV-trnM* | 840 | 1 | 2 |
|  | *trnP-petG* | 488 | 1 | 5 |
|  | *rpL20-rpS12* | 765 | 0 | 2 |
|  | *psbB-psbH* | 587 | 0 | 0 |
|  | *rpL16* | 1031 | 3 | 0 |
| Designed in this study | *ycf1* | 394 | 9 | 0 |
|  | *ndhH-rps15* | 434 | 5 | 1 |
|  | *psbK-psbI* | 446 | 4 | 3 |
|  | *cosA-ndhD* | 469 | 5 | 2 |
|  | *ndhG-ndhI* | 462 | 4 | 3 |

**Table S4** Primers for ITS and plastid sequences

| target | Primer names | sequence | citation |
| --- | --- | --- | --- |
| ITS1-ITS4 | ITS1 | TCCGTAGGTGAACCTGCGG | Mayuzumi & Ohba 2004 |
|  | ITS4 | TCCTCCGCTTATTGATATGC |  |
| *psbA-trnH* | psbAF | GTTATGCATGAACGTAATGCTC | Sang & Crawford 1997 |
|  | trnHR | CGCGCATGGTGGATTCACAAATC |  |
| *ycf1* | ycf1F | GTATTCTTGGTATTAGTCTGGATCCA |  |
|  | ycf1R | GTACCAAAAAAATGGAAAAAGAAGAAAAAT |  |
| *ndhH-rps15* | ndhHF | CATAAGTTCATTTCTTATAATTGGTACATTCAT |  |
|  | rps15R | TCTACCAAATTAAAATGTCCAACATTATTAT |  |
| *psbK-psbI* | psbKF | TCTTATGCCAGTTATACCTCTGC |  |
|  | psbIR | TGTAAACGAAGAGTTTGAGAGTAA |  |
| *cosA-ndhD* | cosAF | TTGTAGCTTCTATGGGCTTTATTATAAT |  |
|  | ndhDR | GATTCGGGACCAAGAGAATTATTT |  |
| *ndhG-ndhI* | ndhGF | AACATTTATAGCTCCCACATAAATAAGAA |  |
|  | ndhIR | TTACACAATTCGAACAATTTTGAATG |  |

**Table S5** SSR loci used in this study

| Loci | Primer names | Sequences | mode |
| --- | --- | --- | --- |
| SSR1 | SSR1F | TCTCCATCAATTCCATGGCCAA | (ACC)n |
|  | SSR1R | ATGTCGAGGAAGAGGAGAGTGA |  |
| SSR2 | SSR2F | ATTCACATGGCTTCTTTGGTGC | (GCC)n |
|  | SSR2R | CCCGACGTCGTTTAGGAAAAAC |  |
| SSR3 | SSR3F | TGGAACATCCTCGGACTGTTTT | (GGA)n |
|  | SSR3R | TACCAACCACTCCACAACCAAA |  |
| SSR4 | SSR4F | AACACTTCGATGTTCTGAGGCT | (GGC)n |
|  | SSR4R | CGTCGTCATCTCTCTGTCTCTG |  |
| SSR5 | SSR5F | TCCCTAAACGTCCTACTCCTCC | (ACG)n |
|  | SSR5R | CCCTCAGAAGCGGAAGATCTAC |  |
| SSR6 | SSR6F | CACCGCTGTTGTTGTTATCTCC | (TTG)n |
|  | SSR6R | GGGAATCTCAACGTCGCTATCT |  |
| SSR7 | SSR7F | CCCTCTTCCCTTCATCATTCCA | (TGG)n |
|  | SSR7R | GTTCGGAACCAGTCTTCGATCT |  |
| SSR8 | SSR8F | GTCGAGTGGGAAGAGATCGAAA | (CTG)n |
|  | SSR8R | TTTGTGTTTGTGCTTCGGATCC |  |
| SSR9 | SSR9F | TCTCCTCGTCCATTTGAACAGG | (AT)n |
|  | SSR9R | AACTGTGTCAAAATGGCGTGTG |  |
| SSR10 | SSR10F | ACGGACGCCATGATGATTGATA | (GA)n |
|  | SSR10R | ACTTTGGGCCTTATCAGTAGCC |  |
| SSR11 | SSR11F | GAGGAGTGATTGGAAGCTGAGT | (GAT)n |
|  | SSR11R | TACTCTTGGACAGCTGCTCAAG |  |
| SSR12 | SSR12F | GTGGAAGGGGTTGGAGTAGAAG | (CT)n |
|  | SSR12R | ATAACACCAAACATGGCAGCAC |  |
| SSR13 | SSR13F | CTCCTTGGACGACATCGATTGA | (CT)n |
|  | SSR13R | ACCCAACCATCTCATACCGAAC |  |

**Fig. S1** Bayesian tree of *Rhodiola* sect. *Prainia* species and related species based on nuclear ribosomal DNA internal transcribed spacer (ITS) sequences constructed by mrbayes 3.2. Nodes are labeled with posterior probabilities.

**Fig. S2** Strict consensus tree of *Rhodiola* sect. *Prainia* species and related species based on nuclear ribosomal DNA internal transcribed spacer (ITS) sequences constructed by paup 4. Nodes are labeled with bootstrap support values based on 1000 replicates.

**Fig. S3** Bayesian tree of *Rhodiola* sect. *Prainia* species and related species based on concatenated chloroplast sequences constructed by mrbayes 3.2. Nodes are labeled with posterior probabilities.

**Fig. S4** Strict consensus tree of *Rhodiola* sect. *Prainia* species and related species based on concatenated chloroplast sequences constructed by paup 4. Nodes are labeled with bootstrap support values based on 1000 replicates.


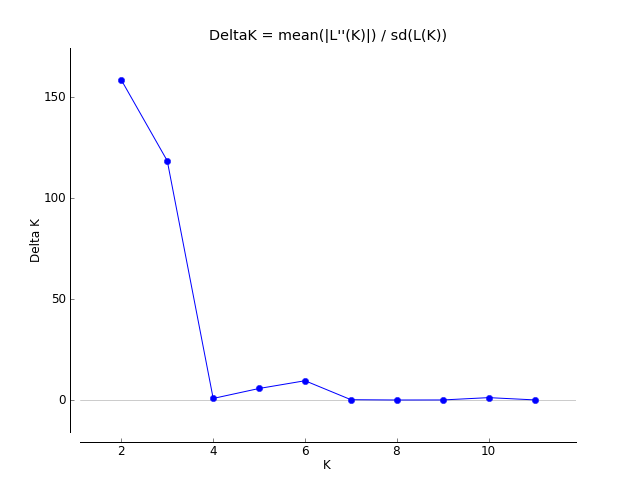


**Fig. S5** Delta K analysis for STRUCTURE results

**Table S6.** Delta K results generated from 10 independent runs in STRUCTUR

| **K** | **Reps** | **Mean LnP(K)** | **Stdev LnP(K)** | **Ln'(K)** | **\|Ln''(K)\|** | **Delta K** |
| --- | --- | --- | --- | --- | --- | --- |
| 1 | 10 | -6532.930000 | 0.163639 | - | - | - |
| 2 | 10 | -4299.940000 | 10.218306 | 2232.990000 | 1617.030000 | 158.248344 |
| 3 | 10 | -3683.980000 | 3.262174 | 615.960000 | 385.970000 | 118.316811 |
| 4 | 10 | -3453.990000 | 95.322347 | 229.990000 | 87.350000 | 0.916364 |
| 5 | 10 | -3311.350000 | 14.755507 | 142.640000 | 86.180000 | 5.840531 |
| 6 | 10 | -3254.890000 | 4.574434 | 56.460000 | 44.210000 | 9.664584 |
| 7 | 10 | -3242.640000 | 44.256555 | 12.250000 | 13.040000 | 0.294646 |
| 8 | 10 | -3217.350000 | 95.907145 | 25.290000 | 14.070000 | 0.146704 |
| 9 | 10 | -3206.130000 | 84.898371 | 11.220000 | 14.640000 | 0.172441 |
| 10 | 10 | -3180.270000 | 39.513262 | 25.860000 | 52.080000 | 1.318038 |
| 11 | 10 | -3206.490000 | 54.530551 | -26.220000 | 10.620000 | 0.194753 |
| 12 | 10 | -3243.330000 | 65.581604 | -36.840000 | - | - |

**Table S7.** Occurrence records of *R. stapfii* and *R. prainii* used in this study.

| Species | Code | Longitude | Latitude | Source | Catalogue |
| --- | --- | --- | --- | --- | --- |
| *R. stapfii* | Rsta1 | 92.345 | 29.029 | pers. obs. | - |
|  | Rsta2 | 92.331 | 29.034 | pers. obs. | - |
|  | Rsta3 | 92.330 | 29.036 | pers. obs. | - |
|  | Rsta4 | 92.328 | 29.035 | pers. obs. | - |
|  | Rsta5 | 92.326 | 29.035 | pers. obs. | - |
|  | Rsta6 | 93.052 | 28.913 | CVH | PE00845582 |
|  | Rsta7 | 92.601 | 28.991 | CVH | PE00845586 |
|  | Rsta8 | 88.82 | 27.759 | CVH | PE00845578 |
|  | Rsta9 | 89.152 | 27.712 | CVH | PE00845581 |
|  | Rsta10 | 91.994 | 28.838 | CVH | PE00055672 |
|  | Rsta11 | 89.086 | 27.742 | CVH | PE00055638 |
|  | Rsta12 | 92.039 | 28.829 | CVH | PE00845580 |
|  | Rsta13 | 92.352 | 29.823 | CVH | PE00845585 |
|  | Rsta14 | 89.2 | 27.68 | PPBC | 1233423 |
|  | Rsta15 | 92.38 | 29.05 | HUP | GMBA-HUP-123436 |
|  | Rsta16 | 89.48 | 27.95 | HUP | GMBA-HUP-173144 |
| *R. prainii* | Rpra1 | 91.07 | 29.67 | HUP | GMBA-HUP-141829 |
|  | Rpra2 | 90.45 | 28.97 | HUP | GMBA-HUP-141562 |
|  | Rpra3 | 91.33 | 30.07 | HUP | GMBA-HUP-174606 |
|  | Rpra4 | 83.695 | 28.710 | HUP | GMBA-HUP-140587 |
|  | Rpra5 | 90.3 | 28.97 | HUP | GMBA-HUP-127900 |
|  | Rpra6 | 90.53 | 28.98 | HUP | GMBA-HUP-136226 |
|  | Rpra7 | 90.98 | 29.73 | HUP | GMBA-HUP-136226 |
|  | Rpra8 | 87.57 | 29.07 | HUP | GMBA-HUP-142891 |
|  | Rpra9 | 91.45 | 29.68 | HUP | GMBA-HUP-122803 |
|  | Rpra10 | 91.15 | 29.75 | HUP | GMBA-HUP-122664 |
|  | Rpra11 | 89.63 | 28.95 | HUP | GMBA-HUP-173142 |
|  | Rpra13 | 86.34 | 28.05 | CVH | PE00845219 |
|  | Rpra14 | 84.85 | 28.66 | CVH | PE01487153 |

Pers. obs.: personal observation in this research; CVH: Chinese Virtual Herbarium (accessed through Chinese Virtual Herbarium(CVH) Data Portal, cvh.ac.cn, 2018-9-3); PPBC: Plant Photo Bank of China (accessed via plantphoto.cn, 2018-9-3); HUP: The Himalayan Uplands Plant Database Version 1 (accessed via GBIF.org, 2018-9-3).

**Table S8.** Correlations between selected factors.

|  | Bio1 | Bio3 | Bio12 | Bio15 |
| --- | --- | --- | --- | --- |
| Bio1 | 1 | 0.343883 | 0.659929 | -0.09472 |
| Bio3 | 0.343883 | 1 | 0.039453 | 0.225931 |
| Bio12 | 0.659929 | 0.039453 | 1 | -0.70165 |
| Bio15 | -0.09472 | 0.225931 | -0.70165 | 1 |
