# Supplementary material for: Quaternary climate change and habitat preference shaped the genetic differentiation and phylogeography of Rhodiola sect. Prainia in the southern Qinghai–Tibetan Plateau
Source: Ecol Evol. 2019 Jun 30;9(14):8305–19. doi: 10.1002/ece3.5406 (PMC6662313; doi:10.1002/ece3.5406)
Supplement: Supplementary file 2 [file ECE3-9-8305-s002.docx]

#To calcuate percentage of private alleles in each population

#sample data

1-1 1 100 103 295 295

1-2 1 103 103 295 290

1-3 1 100 103 295 295

1-4 1 103 103 295 290

1-5 1 100 103 295 295

1-6 1 103 103 295 290

1-7 1 100 103 295 295

1-8 1 103 103 295 290

1-9 1 100 103 295 295

1-10 1 103 103 295 290

1-11 1 100 103 295 295

1-12 1 103 103 295 290

2-1 2 100 100 295 285

2-2 2 106 103 295 285

2-3 2 106 106 285 285

2-4 2 100 100 295 285

2-5 2 106 103 295 285

2-6 2 106 106 285 285

2-7 2 100 100 295 285

2-8 2 106 103 295 285

2-9 2 106 106 285 285

2-10 2 100 100 295 285

2-11 2 106 103 295 -9

2-12 2 106 106 285 285

2-13 2 100 100 295 285

2-2 14 -9 -9 295 285

2-3 15 106 106 285 285

#copy all data to clipboard manually, only works for windows

#Then, read the clipboard using the following codes.

alldata <- read.table(file = 'clipboard')

#set parameters

#set number of populations

populations <- unique(alldata[,2])

npop <- length(populations)

#set number of loci

nloci <- 13

#set missing data sign

misdata <- -9

#for each population, sample n individuals

nindi <- 10

#for each population, repeat n times

nrep <- 100

#define functions

#get a population

getPop <- function(popid){

return(alldata[alldata[,2]==popid,])

}

#exclude a population

exPop <- function(popid){

return(alldata[alldata[,2]!=popid,])

}

#select n random samples in a population

selectRandomIndividual <- function(popid, n){

tempdata <- getPop(popid = popid)

popsize <- nrow(tempdata)

sampledata <- tempdata[sample(1:popsize,n),]

return(sampledata)

}

#get all the alleles, given a pop, and a loci

getAllele <- function(mdata,loci){

temp <- unique(unlist(mdata[,(2*loci+1):(2*loci+2)]))

if(sum(grepl(-9,temp))){

temp <- temp[-which(temp==-9)]

}

return(temp)

}

#calculate allele number, given an allele, a loci, a data matrix

getAlleleN <- function(allele, loci, mdata){

return(sum(mdata[,(2*loci+1):(2*loci+2)]==allele))

}

#Major scripts: calculate percentage of private allele

#to store results

presults <- matrix(nrow = nrep, ncol = npop)

#for each population

for(i in populations){

#start n reps

for(j in 1:nrep){

#get metapopulation, and generate sampled target population

metapopulation <- exPop(i)

samplepopulation <- selectRandomIndividual(i, nindi)

#to store results of each loci

lociresults <- vector(mode = 'numeric', length = nloci)

#for each loci

for(k in 1:nloci){

#get all allele, and sum

allAllele <- getAllele(samplepopulation, k)

allelen <- length(allAllele)

#for each allele, test if it is private

tempfreq <- 0

for(m in 1:allelen){

#if foundoutside is 0, is not found outside, is private

foundOutside <- sum(grepl(allAllele[m], metapopulation[,(k*2+1):(k*2+2)]))

#if it is private, add it to tempfreq

if(foundOutside == 0){

tempfreq <- tempfreq + sum(grepl(allAllele[m], unlist(samplepopulation[,(k*2+1):(k*2+2)])))/(nindi*2-sum(grepl(-9, unlist(samplepopulation[,(k*2+1):(k*2+2)]))))

}

#end each allele

}

lociresults[k] <- tempfreq

#end each loci

}

presults[j,which(populations == i)] <- mean(lociresults)

#end n reps

}

#end each population

}

#show results of each run

presults
